# MADM-ML, a Mouse Genetic Mosaic System with Increased Clonal Efficiency

**DOI:** 10.1371/journal.pone.0077672

**Published:** 2013-10-15

**Authors:** Astra Henner, P. Britten Ventura, Ying Jiang, Hui Zong

**Affiliations:** Institute of Molecular Biology, University of Oregon, Eugene, Oregon, United States of America; University of Maastricht (UM), Netherlands

## Abstract

Mosaic Analysis with Double Markers (MADM) is a mouse genetic system that allows simultaneous gene knockout and fluorescent labeling of sparse, clonally-related cells within an otherwise normal mouse, thereby circumventing embryonic lethality problems and providing single-cell resolution for phenotypic analysis *in vivo*. The clonal efficiency of MADM is intrinsically low because it relies on Cre/loxP-mediated mitotic recombination *between* two homologous chromosomes rather than within the same chromosome, as in the case of conditional knockout (CKO). Although sparse labeling enhances *in vivo* resolution, the original MADM labels too few or even no cells when a low-expressing Cre transgene is used or a small population of cells is studied. Recently, we described the usage of a new system, MADM-ML, which contains three mutually exclusive, self-recognizing loxP variant sites as opposed to a single loxP site present in the original MADM system (referred to as MADM-SL in this paper). Here we carefully compared the recombination efficiency between MADM-SL and MADM-ML using the same Cre transgene, and found that the new system labels significantly more cells than the original system does. When we established mouse medulloblastoma models with both the original and the new MADM systems, we found that, while the MADM-SL model suffered from varied tumor progression and incomplete penetrance, the MADM-ML model had consistent tumor progression and full penetrance of tumor formation. Therefore MADM-ML, with its higher recombination efficiency, will broaden the applicability of MADM for studying many biological questions including normal development and disease modeling at cellular resolution *in vivo*.

## Introduction

**Figure 1:**
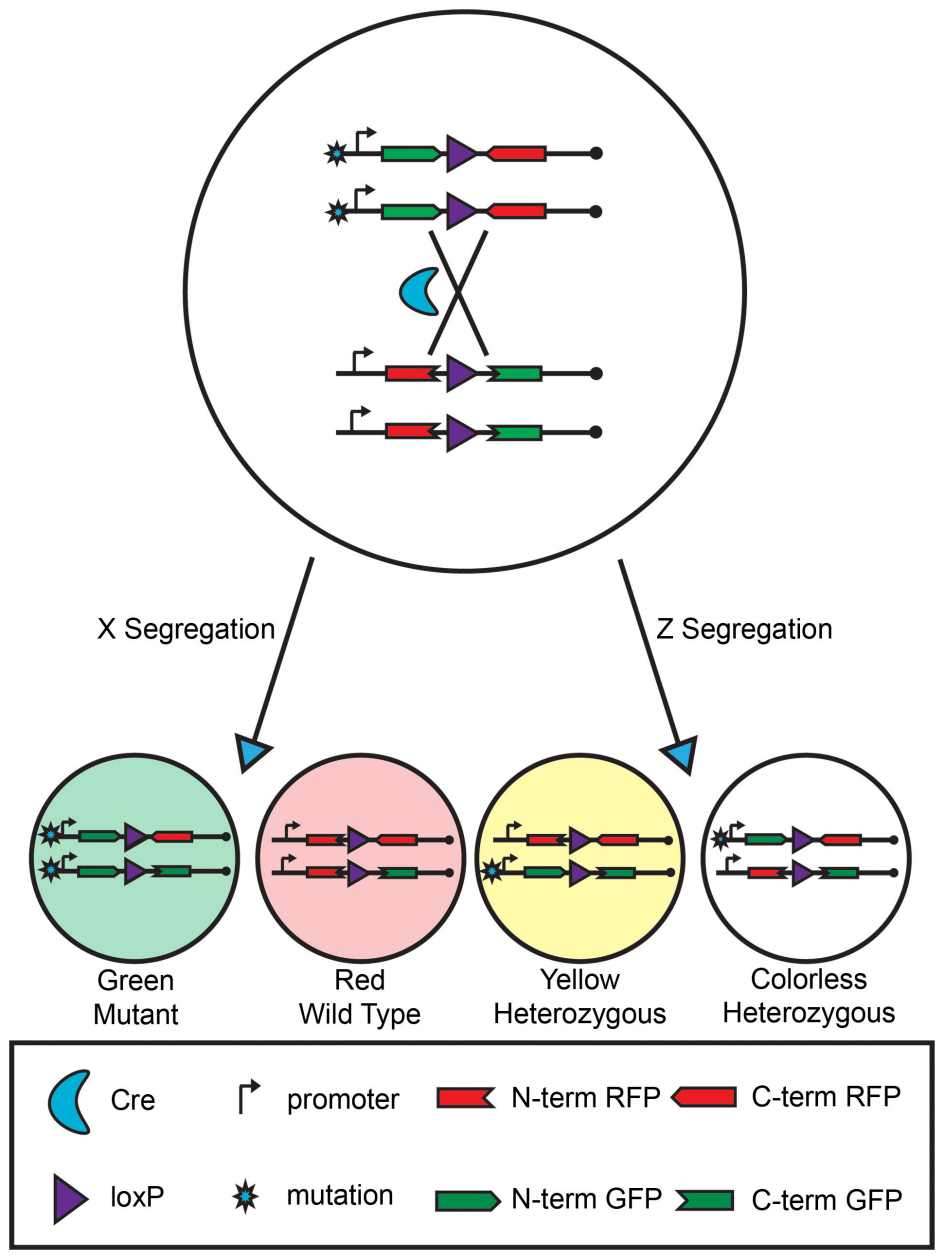
The MADM system. MADM generates sibling green mutant and red wildtype daughter cells from a colorless heterozygous progenitor via Cre-loxP mediated mitotic recombination followed by X segregation. MADM can also generate yellow and colorless heterozygous cells when mitotic recombination is followed by Z segregation.

 Many human diseases, including cancer and some neurodegenerative diseases, are a consequence of genetic mosaicism, i.e., the pathogenic cells contain modified genomes compared to the normal cells in the body. To gain insights into aberrations occurring at the single cell level during pathogenic processes, we created a mouse genetic mosaic system termed MADM that allows simultaneous gene knockout and fluorescent labeling of sparsely distributed cells within an otherwise normal animal [1]. The capability of generating a small number of fluorescently labeled mutant cells allows one to study cell-autonomous gene functions and developmental lineage relationships with single-cell resolution *in vivo*. By design, MADM generates a pair of GFP and RFP labeled daughter cells from a colorless progenitor cell via Cre/loxP-mediated mitotic recombination between two homologous chromosomes followed by G2-X type chromatid segregation (Figure 1). When a mutant allele of a gene of interest is located at the telomeric side of one of the MADM cassettes as depicted in Figure 1, the green cell will be homozygous mutant while the red cell will be wild type (WT) for that gene. Due to the sibling relationship with the green mutant cell, the red WT cell serves as an ideal internal control for *in vivo* phenotypic analysis at subcellular resolution, such as neuronal morphogenesis defects (illustrated in Figure S1A). Importantly, since the cell labeling is permanent, one could analyze lineage development for both GFP- and RFP-labeled cells and reveal specific genetic contributions to normal development and disease onset (illustrated in Figure S1B). It should be noted that, if an alternative type of segregation, called G2-Z occurs after the mitotic recombination, one yellow and one colorless cell will be produced, both of which are still heterozygous for the gene of interest (Figure 1) and are relatively less useful for phenotypic analysis. 

 With these well-designed features, the MADM system has helped answer some important questions in developmental biology, neurosciences, and cancer biology. For example, MADM was first used to study normal cerebellar development, in particular to examine the relationship between lineage and axonal wiring of granule cells [1] and to analyze cell division patterns in the granule neuron lineage [2]. Later MADM has also been applied to study genetic control of neuronal morphogenesis, migration and proliferation/survival [2-4], cell cycle regulation [5], the identification of the cell of origin for glioma [6], and the effects of loss of imprinting (LOI) in somatic cells [7].

 Although the sparseness of MADM labeling is an advantage for the aforementioned studies, the rarity of labeled cells precludes other applications. Previously, we found that Cre/loxP mediated recombination varies greatly depending on the Cre transgenes used [1]. Of the nine Cre lines tested, the labeling efficiency of the most potent Cre lines was approximately 1-2% of total cells while the efficiency of weaker Cre lines was orders of magnitude lower. Most strikingly, a commonly used germline deleter Cre line, β-actin-Cre, had extremely low labeling efficiency with MADM [1]. Since β-actin-Cre has been well known to be 100% effective for conditional knockouts (CKOs) when both loxP sites on the same chromosome [8], it suggests that inter-chromosomal recombination in MADM requires much higher levels of Cre activity than intra-chromosomal recombination in CKO does. Because the cell-type specificity of Cre transgenes is of high priority for studies to reach definitive conclusions, the expression level of many available Cre transgenic lines tends to be relatively low. Additionally, CreER lines that are highly valuable for temporal analysis of normal development or disease progression tend to have lower recombination activity even when used in CKO models. Therefore, in both cases, one would not be able to generate sufficient number of labeled cells in MADM for phenotypic analysis, thereby greatly limiting the applicability of MADM. Therefore, it is very important to establish an optimized MADM system with higher recombination efficiency for broader applications. 

 Recently, we reported the use of a modified MADM system could have higher recombination efficiency [6,7,9]. In the new system, we replaced the original single loxP site with multiple self-recognizing but mutually exclusive loxP variants (herein we refer to the original MADM system as MADM-SL for ‘single lox’ and the new system as MADM-ML for ‘multiple lox’). In this study, we report the detailed characterization of MADM-ML in comparison to MADM-SL based on the cell labeling efficiency in cerebellar granule neuron precursors (GNPs). We found that MADM-ML significantly increases recombination efficiency, without negative impact on G2-X segregation in all labeled cells. We also tested the utility of the MADM-ML system by comparing medulloblastoma models established with both the original MADM-SL and the new MADM-ML system. We found that while the MADM-SL based tumor model had incomplete penetrance and an inconsistent time course of tumor progression, the MADM-ML based tumor model was free of both issues. Therefore, the newly established MADM-ML system is a valuable mouse genetic tool and should have broad application in many research areas, such as developmental biology, neurobiology, and disease modeling.

## Results

### The establishment of a MADM system with multiple loxP variants

 The inter-chromosomal recombination efficiency of MADM is determined by the concentration of three molecules, Cre recombinase and two homologous chromosomes, each containing a loxP site. Since we could neither modulate the number of homologous chromosomes (one pair per cell) nor change the protein concentration of Cre recombinase in a given transgenic line, the only way to enhance the reaction rate relies on introducing more loxP sites into each chromosome. A string of wildtype loxP sites would not achieve this goal since they could recombine with each other *in cis* and *in trans* in the presence of Cre, leaving only one loxP site after reactions cease. Therefore, we decided to replace the single loxP site with multiple self-compatible but mutually exclusive loxP variant sites. 

**Figure 2:**
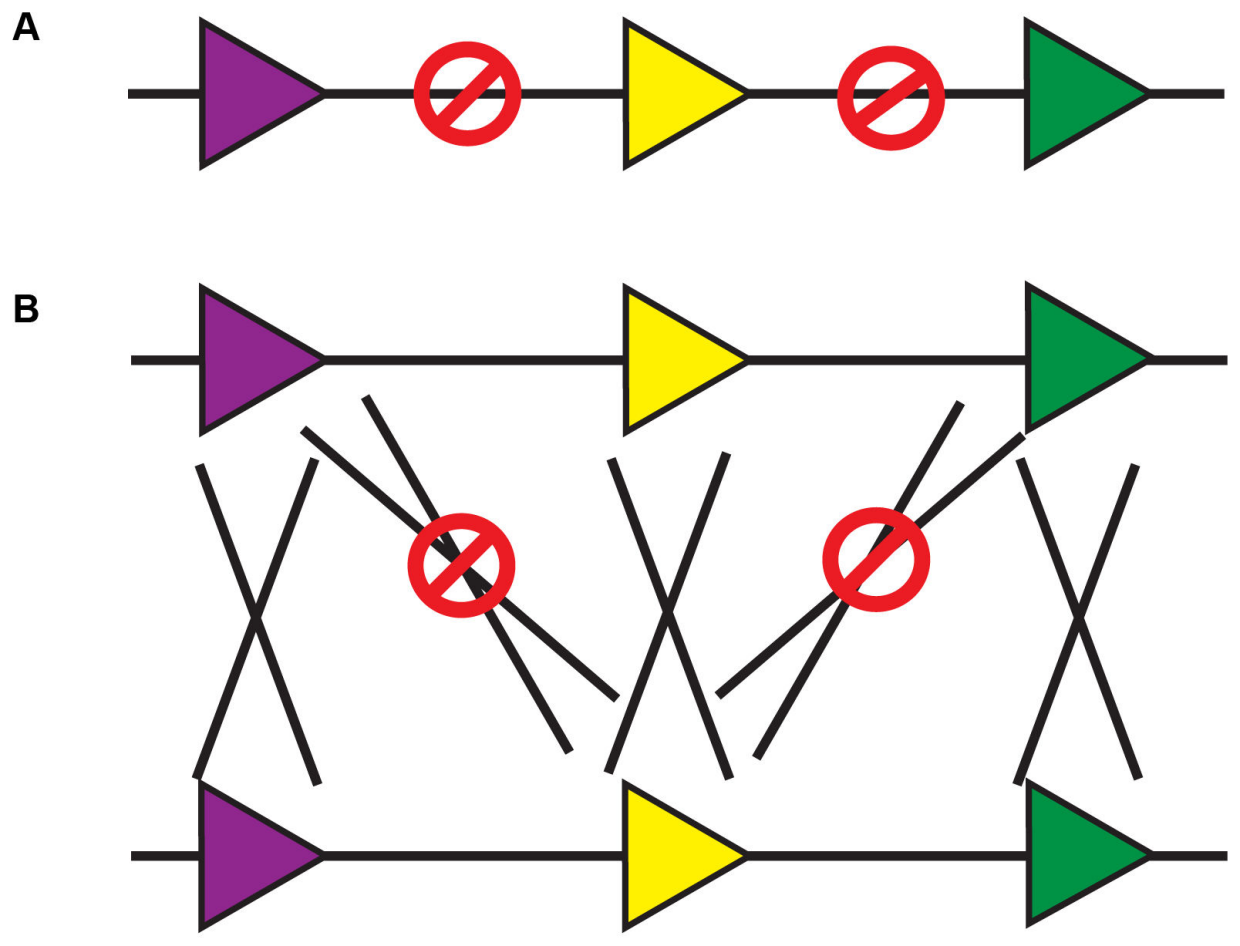
The design of MADM with multiple loxP sites (MADM-ML). (A) Mutually exclusive loxP sites (wildtype loxP and loxP variants lox2272 and lox5171) do not recombine with each other *in* *cis*. (B) These loxP variants recombine *in* *trans*, but only with their identical sites, but not with other variants.

 LoxP is a 34 bp sequence that contains an 8-basepair asymmetric core sequence flanked by two sets of 13-basepair palindromic sequences. Previously, mutagenesis studies of the loxP core sequence yielded several loxP variants that could self-recombine but not cross-react with WT or other variant loxP sites [10]. Although none of the variants recombined as efficiently as WT loxP, variants lox2272 and lox5171 showed maximal recombination efficiency with themselves and minimal promiscuity with each other or with a WT loxP site [10]. Based on these considerations, we replaced the single loxP site in the original MADM cassette on chromosome 11 [3] with a string of three loxP variants, including WT-loxP, lox2272, and lox5171 at the same *hipp11* locus, MGI:5461148 (Figure 2 and see Materials and Methods section for details). By design, these loxP variants on the same chromosome should not be able to recombine with each other *in cis* (Figure 2A), but should be able to recombine *in trans* with their respective identical sites on the homologous chromosome (Figure 2B), resulting in an augmented recombination rate without the risk of losing loxP sites. We named the new MADM system MADM-ML (multiple lox), and referred to the original system as MADM-SL (single lox) in this paper. 

### Increased recombination efficiency and unaffected G2-X segregation with MADM-ML

 To evaluate the effectiveness of the multiple loxP sites, we examined two important parameters of MADM-ML, the overall recombination efficiency and the percentage of G2-X chromosome segregation pattern. The latter is important because only the G2-X segregation produces green mutant and red WT cells while the G2-Z segregation produces double-labeled heterozygous cells that are not as useful for phenotypic analysis. 

 To compare these parameters between MADM-SL and MADM-ML, we generated MADM mice with the Math1-Cre (also known as Atoh1-Cre) transgene [11]. We chose Math1-Cre because it is specifically expressed in granule neuron precursors (GNPs) in the developing cerebellum, which has easily defined locations for the quantification of labeled cells [11]. GNPs are derived from neural stem cells in the fourth ventricle early in development. Starting at E12 in mice, a cohort of stem cells migrates through a structure known as the rhombic lip, populates the surface of the cerebellar primordium known as the external germinal layer (EGL), and becomes committed GNPs [12]. Postnatally, GNPs go through an exponential expansion phase, then exit the cell cycle and migrate inward past the molecular layer to populate the inner granule layer (IGL), where they differentiate into granule neurons. Due to their exceptional proliferative capacity, deregulated GNP growth results in medulloblastoma formation [13]. 

**Figure 3:**
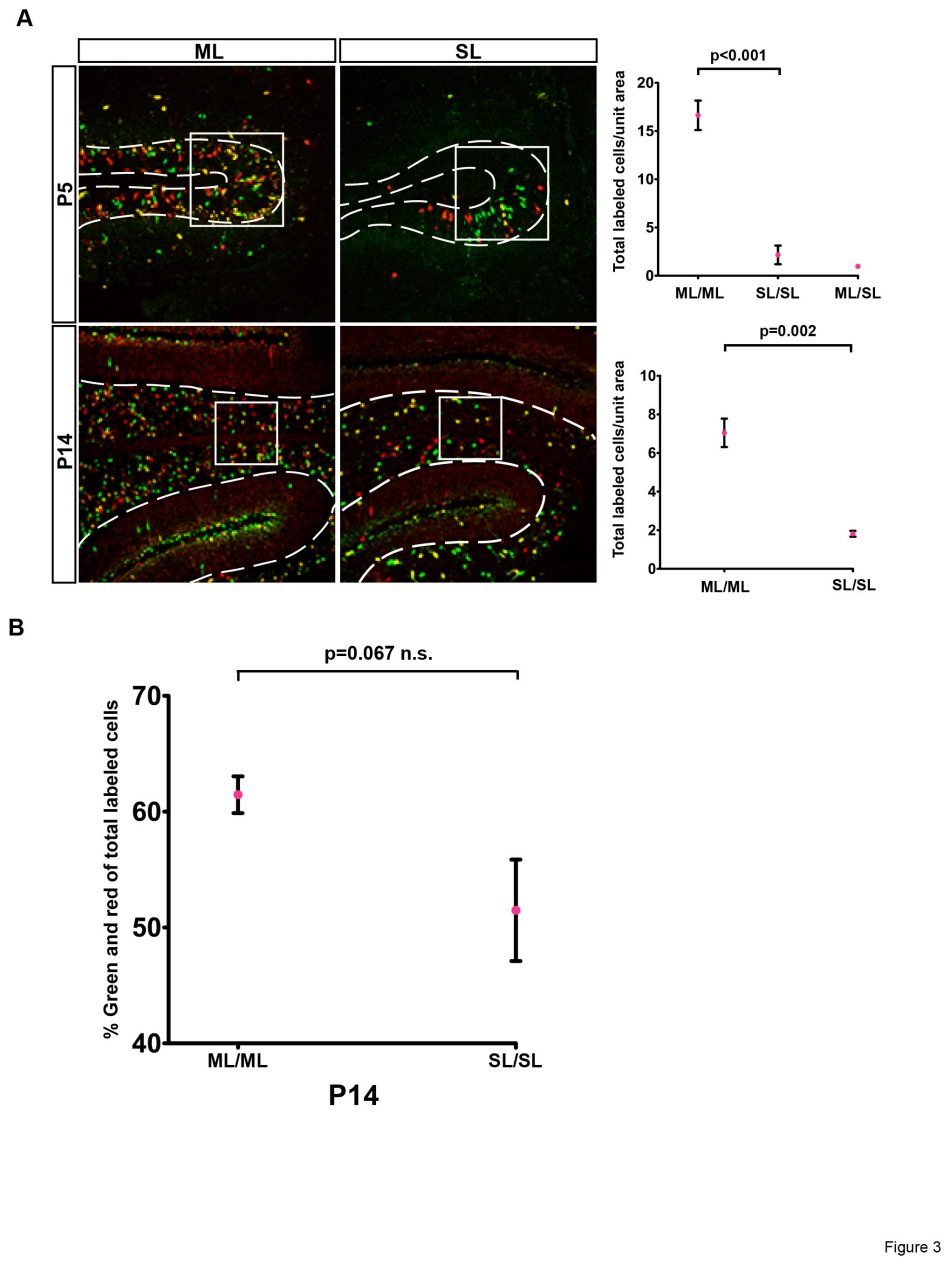
MADM-ML has significantly higher recombination efficiency than MADM-SL, and maintains the productive G2-X segregation pattern. (A) Representative images of cerebella in MADM Math1-Cre mice at P5 and P14 with quantification areas outlined (EGL for P5, IGL for P14). MADM-ML has significantly more labeled cells than MADM-SL does, for both P5 and P14 data set. P5 data (n=5 for ML/ML, n=4 for SL/SL, and n=5 ML/SL) are analyzed by ANOVA and multiple comparisons with Tukey HSD. There is no significant difference between SL/SL and ML/SL. P14 data (n=5 for ML/ML and n=7 for SL/SL) are analyzed by an independent samples t-test with unequal variance. (B) There is no significant difference in G2-X segregation frequency between the two MADM systems, as measured by percent of green and red cells among total labeled cells. n=5 for ML/ML and n=7 for SL/SL. Error bars are one standard deviation.

 For quantification analysis, we collected cerebella at two ages, P5 (postnatal day 5) when the majority of GNPs are actively proliferating in the EGL, and P14 when most GNPs migrate into the IGL and differentiate into granule neurons. All mice analyzed were heterozygous for the Math1-Cre transgene so any differences in recombination efficiency would be attributed to the number of loxP sites rather than the varied level of Cre expression. Based on the developmental process, we counted all labeled cells within the appropriate cerebellar region according to the age of samples, EGL for P5 and IGL for P14. To establish a reliable counting scheme, we carefully examined many brain sections at these ages to look for regions of consistent labeling, and chose the EGL at the base of the sulcus between the fifth and sixth folia in P5 brains (Figure 3A, top panels), and the base of the third folium within the IGL for P14 brains (Figure 3A, bottom panels). To ensure systematic sampling, three brain slices from different slides at similar medial-lateral positions were analyzed, and at least four mice per group were used for analysis (see Materials and Methods for more details). The quantification of total numbers of labeled cells (green, red, and yellow) showed that MADM-ML had a 4-8 fold increase compared to MADM-SL (Figure 3A), suggesting a significant improvement of recombination efficiency due to the addition of two loxP variants into the original MADM system. To determine the rate of productive G2-X segregation, we calculated the percentage of green and red cells among all labeled cells. The G2-X percentage was approximately 50% using the MADM-SL system and 60% with MADM-ML, a statistically insignificant difference. These results demonstrated that adding a string of loxP variants in MADM-ML led to a significant increase in recombination efficiency without negatively impacting the rate of G2-X segregation, validating the use of MADM-ML for applications requiring increased cell labeling.

### Increased recombination efficiency is likely due to increased loxP-site pairing

**Figure 4:**
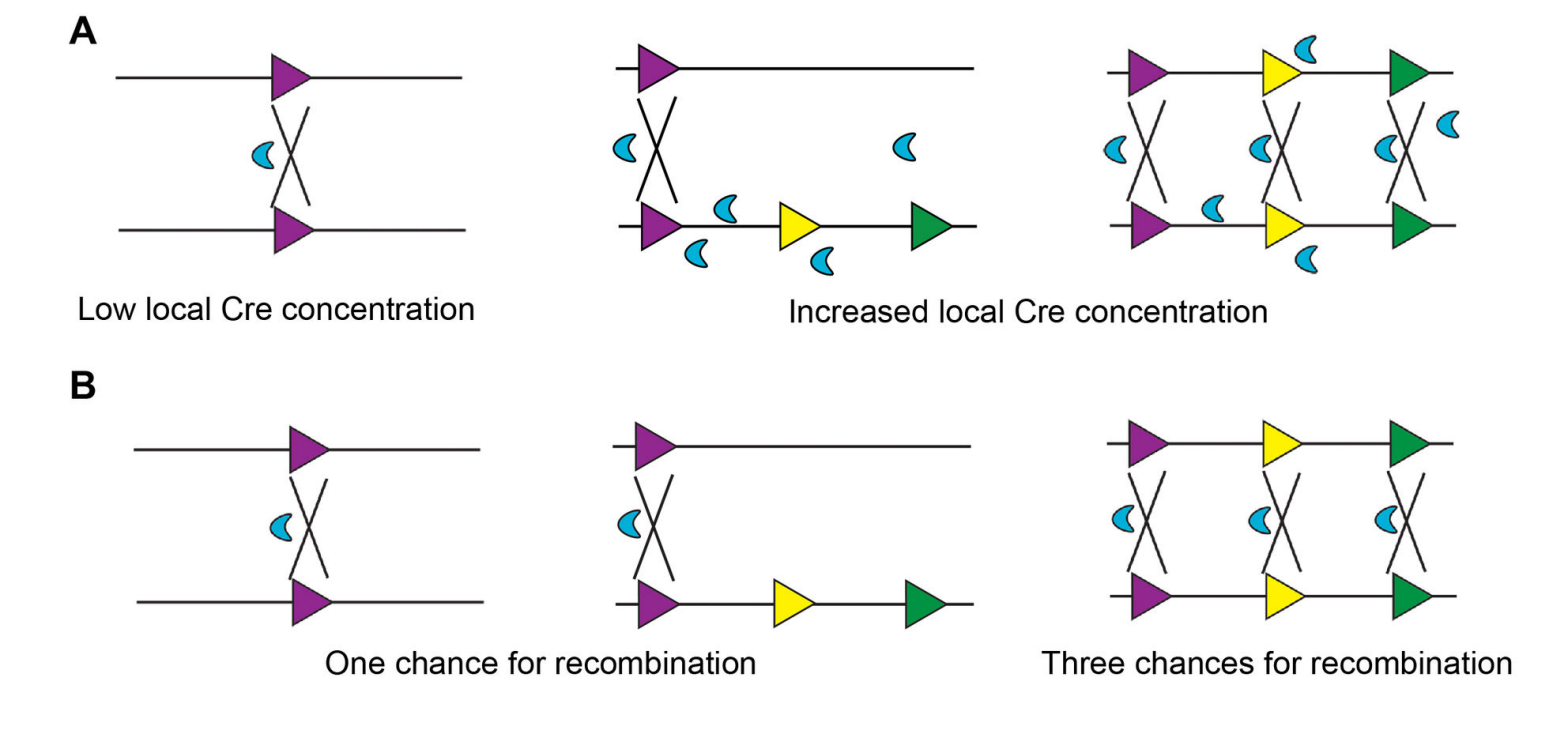
Two possible mechanisms for increased recombination efficiency of MADM-ML. (A) Hypothesis #1: increased recombination efficiency could result from higher local Cre recombinase concentration retained by multiple loxP sites. If this is the case, then ML/SL should have an intermediate recombination efficiency between SL/SL and ML/ML. (B) Hypothesis #2: increased recombination efficiency could be due to increased substrate concentration, i.e. three times more loxP site pairing. If this is the case, then ML/SL should have the same recombination efficiency as SL/SL has.

 The increased recombination efficiency in MADM-ML could be explained by two hypotheses. The first hypothesis is that the presence of multiple loxP sites increases the local Cre concentration at the steady state, thereby enhancing recombination efficiency (Figure 4A). The second hypothesis is based on simple probability, considering that more loxP sites would result in increased opportunities for recombination (Figure 4B). Distinguishing between these hypotheses is very important to guide the utility of the MADM-ML system, particularly whether or not one could mix ML and SL alleles to achieve intermediate recombination efficiency. If the first hypothesis is correct, we should be able to observe intermediate recombination efficiency when one MADM allele contains multiple loxP variants while the other contains only a single loxP site (ML/SL). However, if the second hypothesis is correct, then the pairing of MADM-ML allele with a MADM-SL allele would result in no improvement from the original MADM-SL.

 To test these hypotheses, we compared the recombination efficiency in three scenarios: two alleles of single loxP (SL/SL), one allele of multiple loxP and one allele of single loxP mice (ML/SL), and two alleles of multiple loxP (ML/ML). Because the data clearly showed that there is no significant difference in labeling efficiency between SL/SL and ML/SL (graph of P5 in Figure 3A), we concluded that the second hypothesis is likely the correct one. In other words, the availability of loxP sites is rate-limiting for inter-chromosomal recombination. Taken together, both MADM-ML alleles should be used when higher recombination is needed, and the combination of MADM-ML and MADM-SL alleles is not recommended.

### Modeling medulloblastoma with MADM-SL

 Medulloblastoma is the most common malignant pediatric brain tumor with a peak occurrence between 3 and 9 years of age [14]. The subtype of medulloblastoma with mutations in Shh pathway components, such as a negative regulator *PTCH1*, arises from cerebellar GNPs [15]. Human genetics studies found that individuals with Gorlin syndrome (germline *PTCH1* mutation) have greatly increased incidences of medulloblastoma before age 5 [16,17]. In addition to *PTCH1* mutation, a cohort of human tumor analysis revealed that medulloblastoma patients with *p53* mutations have a much worse prognosis compared to those with wildtype *p53* gene, strongly suggesting its involvement in tumor progression [18]. In mouse models, it was found that while medulloblastoma in a heterozygous *Ptc* knockout model occurred at ~15% penetrance with long latency, a mouse model that combined *p53* mutation with a *Ptc* heterozygous background generated medulloblastoma at almost full penetrance with a much shorter latency [13,19].

**Figure 5:**
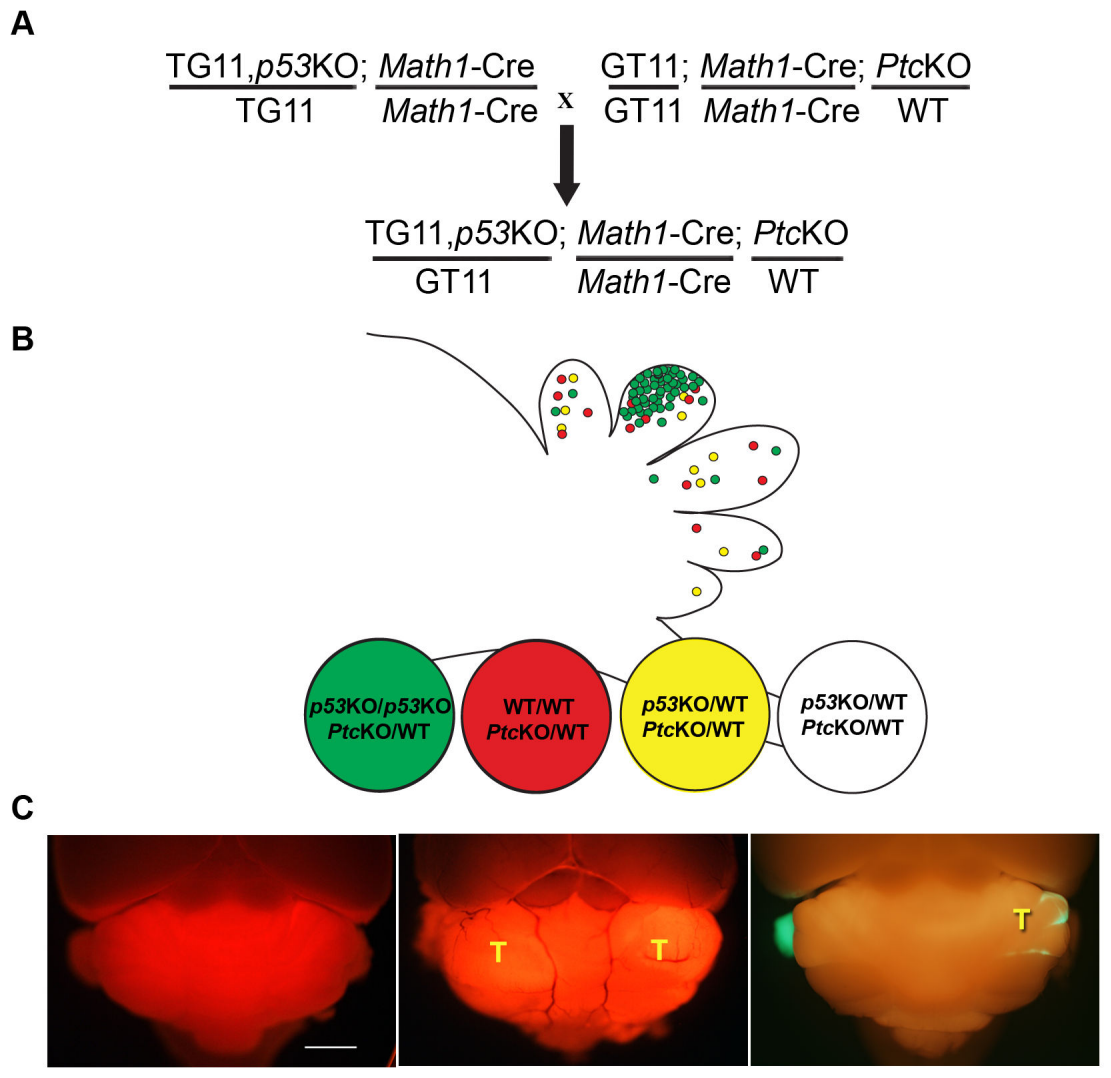
The generation of a medulloblastoma model with the MADM system. (A) Breeding scheme for generating medulloblastoma mouse model. (B) The correlation between fluorescent protein labeling and genotype of cells generated by MADM in this model. All cells in the animal are heterozygous for *Ptc* mutation but *p53* status varies. Green cells are homozygous null for *p53*; red cells are homozygous WT for *p53*; yellow and colorless cells are heterozygous for *p53* (KO/WT). (C) Left, a normal adult brain contains a floxed-stop Rosa-tdTomato Cre reporter and *Math1*-Cre. Middle, CKO medulloblastoma model contains the same Cre line and reporter, heterozygous *Ptc* mutation, and homozygous *p53flox* alleles. Right, an early tumor formed using our MADM mudulloblastoma model. T: tumor. Scale bar: 500um.

 Based on these findings, we established a MADM-SL based medulloblastoma model by using Math1-Cre [11] to specifically inactivate *p53* in a small number of GNPs in a heterozygous *Ptc* background [20] (Figure 5A,B). Although the initial labeling efficiency was relatively low (Figure S2), medulloblastoma did arise in our model (Figure 5C right panel). The sparse labeling of cells in the MADM model provides great *in vivo* resolution, which allows us to identify small tumors that could be easily missed with conventional CKO models. As an example, in a conventional CKO model of medulloblastoma with a floxed-stop tdTomato Cre reporter, small lesions are difficult to see because all differentiated granule neurons in the IGL become labeled in addition to tumor cells (Figure 5C. left panel: no tumor; middle panel, tumor). On the contrary, MADM allows us to easily locate very small lesions (Figure 5C right panel). 

**Figure 6:**
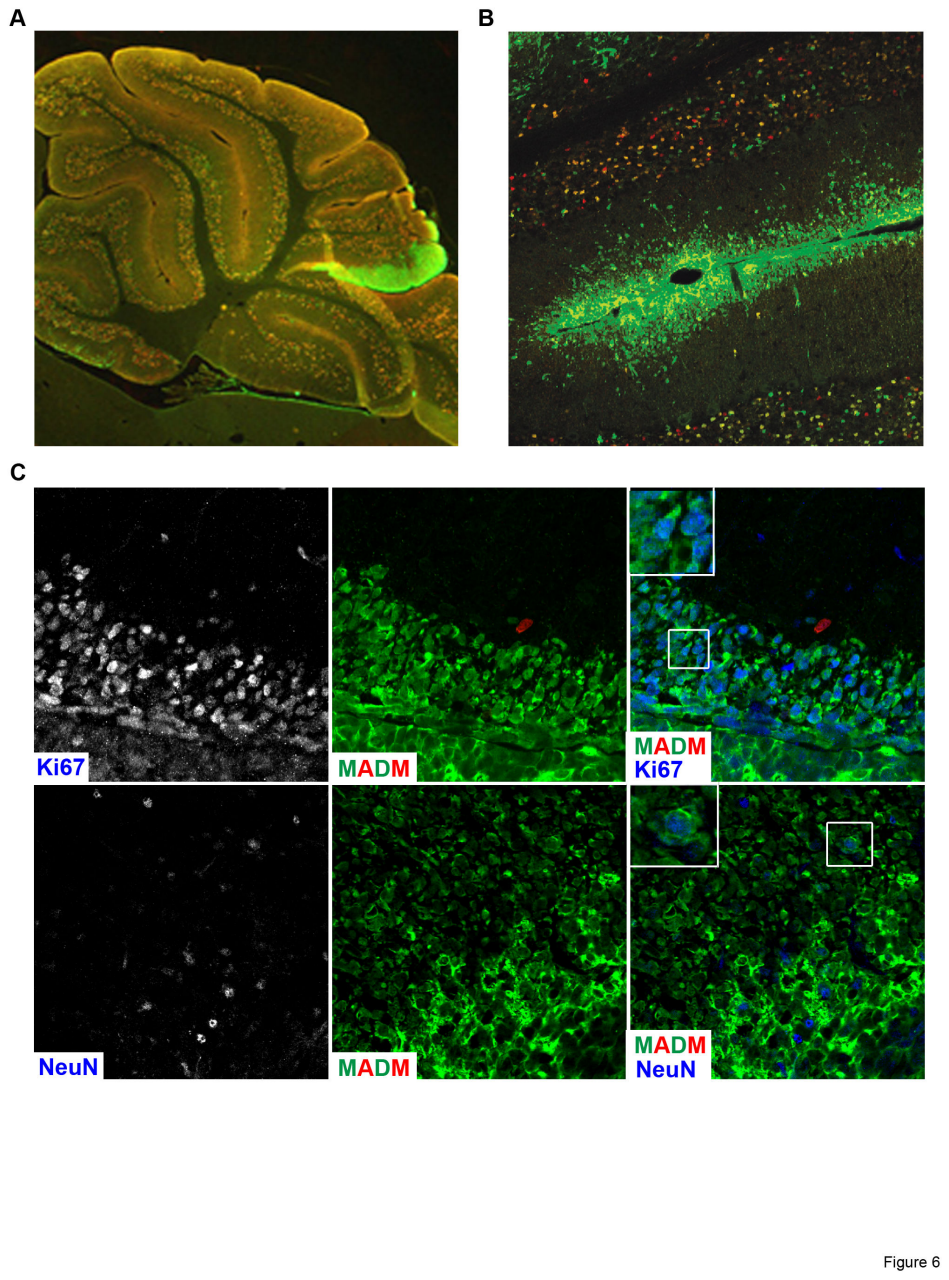
MADM allows visualization of early lesions and detailed tumor organization. (A) A representative brain section from a MADM-generated medulloblastoma. The GFP-labeling of mutant cells amidst sparse background labeling of granule neurons clearly identifies early lesion sites consisting of only hundreds to thousands of cells in a P90 mouse. (B) Tumor cells in the small lesion are GFP+, suggesting that p53 loss is critical for malignant transformation. (C) Co-staining of GFP with proliferation or differentiation markers allows us to perform detailed analysis of tumor organization. Top panels show many actively dividing tumor cells that are Ki67+ GFP+ (inset is a zoomed-in image showing clear GFP/Ki67 overlap). Bottom panels show NeuN+ GFP+ cells within the tumor mass, suggesting that some tumor cells differentiate into neurons (inset is a zoomed-in image showing clear GFP/NeuN overlap).

 To investigate whether or not all tumor cells originated from *p53*-null cells, we analyzed more than 10 brains containing MADM-induced medulloblastoma. We found that tumor cells are almost exclusively green (Figure 6A, B), suggesting that the loss of *p53* is a critical transformation event that leads to medulloblastoma in this model. Previously, it was reported that neuronal markers such as NeuN are often found within the tumor mass of medulloblastoma. However, it was not known if those NeuN+ cells are differentiated from tumor cells or simply entrapped normal neurons. This is another question conventional CKO models cannot easily answer because all cells from GNP-lineage are labeled, including both the tumor cells and the normal granule neurons. With the MADM system, only granule neurons derived from GFP+ mutant cells will remain green while the vast majority of heterozygous granule neurons will not be labeled. Therefore, GFP- NeuN+ cells in the tumor mass should be entrapped WT neurons, while GFP+ NeuN+ neurons should be differentiated from tumor cells. When we examined tumor sections from the MADM-based medulloblastoma model (Figure 6A, B), we found not only GFP+ Ki67+ dividing tumor cells (Figure 6C, upper panel) but also many GFP+ NeuN+ cells within the tumor mass (Figure 6C, lower panel). This finding suggests that even malignant tumor cells retain the tendency to differentiate into neurons, which could be exploited to develop differentiation therapy for medulloblastoma patients.

### MADM-ML based medulloblastoma model has full tumor penetrance, shorter latency, and relatively consistent tumor progression

 One important application of mouse cancer models is to test the efficacy of drugs in an *in vivo* setting. To make the evaluation reliable, an ideal mouse cancer model should have high penetrance of tumorigenesis and relatively consistent timeline of tumor progression. While the MADM-SL based medulloblastoma model provided some interesting insights toward tumor progression, we found that tumor penetrance was quite low, most likely due to the low number of initial mutant cells. Since inconsistency in tumor incidences makes the model unsuitable for preclinical drug testing, we first attempted to improve tumor penetrance by doubling the Cre dosage with homozygous Math1-Cre. While this change led to a shorter latency (reduced from ~90 days to ~60 days), no improvement in tumor penetrance was observed (30 tumors out of 66 mice with one copy of Cre; 20 tumors out of 41 mice with two copies of Cre). As previously reported, homozygous β-actin-Cre had ~2-fold increase of recombination efficiency when compared to heterozygous Cre [1]. Therefore, the observed latency shortening is probably due to a moderate increase in the number of *p53* null cells in the homozygous Math1-Cre mice. However, a small increase in recombination efficiency by doubling the Cre dosage is not enough to have a significant impact on tumor penetrance.

**Figure 7:**
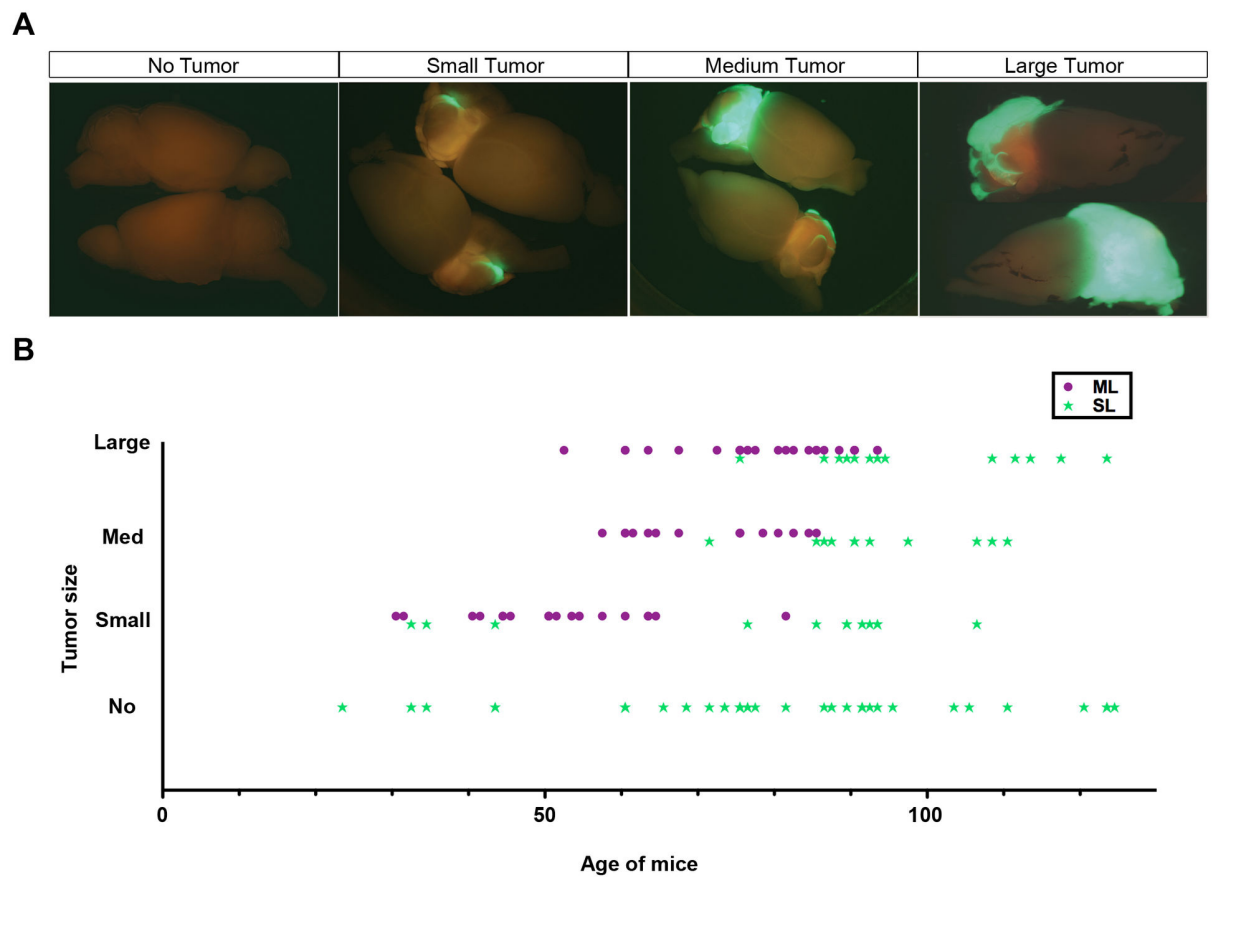
MADM-ML medulloblastoma model generates tumors at full penetrance with a more consistent timeline of tumor progression. (A) Representative images used for scoring tumor size and penetrance. (B) Summary of different tumor sizes in MADM-SL (green stars) and MADM-ML (purple dots) models at various ages. MADM-SL does not have a predictable tumor progression time course and has less than 40% tumor penetrance even after P75. Conversely, MADM-ML has a relatively consistent timeline of tumor progression and full penetrance.

 Since the MADM-ML system was shown to have significantly increased labeling efficiency compared to MADM-SL, we next established a medulloblastoma model with MADM-ML. To compare tumor penetrance, latency and progression in our two MADM systems, we analyzed tumor sizes at a variety of ages based on the size of GFP-positive tumor masses on the cerebellar surface (see representative images depicted in Figure 7A). We scored tumors as “large” if they covered more than half of the cerebellum, “medium” if they covered less than half but more than approximately a third of the cerebellum, and “small” if the tumors covered less than a third. While the MADM-SL model showed low penetrance, long latency, and great inconsistency of tumor progression, the MADM-ML model showed full penetrance, short latency, and a relatively consistent tumor progression (Figure 7B). Therefore, the enhanced recombination efficiency of MADM-ML led to a medulloblastoma model that is much more suitable for applications where consistent tumor progression is desirable.

## Discussion

Gene knockout is a powerful technique for studying gene function in an *in vivo* setting. However, the conventional knockout technique, which generate animals with homozygous mutations in every cell in the organism, frequently result in embryonic lethality that precludes insights into gene functions in later developmental stages and adulthood. By generating sparsely distributed, fluorescently labeled cells with homozygous gene mutations in an otherwise normal mouse, MADM enables phenotypic analysis *in vivo* at single-cell resolution while circumventing the embryonic lethality problem in conventional knockout mice. Since MADM generates a pair of differentially labeled sibling cells, with one cell being mutant and the other WT, it provides a robust internal control for robust phenotypic analysis *in vivo*. In addition, the permanent labeling by MADM enables lineage tracing of mutant and WT cells for the analysis of mutational impacts on developmental processes. It also allows the study of the complete process of pathogenesis, which was applied in our lab to identify the cell of origin in glioma. We introduced oncogenic mutations into neural stem cells (NSCs), and unexpectedly identified oligodendrocyte precursor cells rather than NSCs as the population that over-expands in response to mutations and serves as the cell of origin of this devastating cancer [6].

 The efficiency of MADM-mediated recombination is low because it’s a tri-molecular reaction that involves the Cre protein and loxP sites present on two homologous chromosomes. Increasing the concentration of any of these components should improve the recombination efficiency. However, augmenting the level of Cre expression is not always possible. In the case of the tamoxifen-inducible, CreER or CreERT2 lines, the recombination efficiency is generally low even for CKO due to the dosage limitation of Tamoxifen. As increasing the number of homologous chromosomes is not feasible either, we reasoned that the only way to improve recombination efficiency would be to replace the single loxP site in the original MADM with multiple loxP sites. To avoid recombination *in cis* or *in trans* that will result in the loss of loxP sites, we added two self-recognizing but mutually exclusive loxP variants [10] next to the original loxP site and created the MADM-ML system. While both lox variants only had 50~60% recombination efficiency in *in vitro* testing [10], to our pleasant surprise, the rate of cell labeling of MADM-ML shot up 4-8 fold when compared to that of MADM-SL. This observation suggests that the impact of multiple loxP sites on inter-chromosomal recombination is not linear, and that further addition of loxP sites could result in further significant increase of the efficiency of MADM. The increased recombination efficiency in MADM-ML could be attributed to either increased concentration of the substrate loxP sites or elevated local Cre concentration in the vicinity of the multiple-loxP-containing chromosomes. Our experiments, which show that the recombination efficiency between one MADM-ML and one MADM-SL alleles is comparable to the recombination frequency between two MADM-SL alleles, support the former possibility. Therefore, whenever higher recombination efficiency is desired, both MADM alleles should be of the ‘ML’-type.

 Previously, Liu et al showed increased inter-chromosomal recombination in mouse embryonic stem (ES) cells when three loxP variants in a string were used [21]. However, with the increase in recombination efficiency they saw a 35% decrease in the productive G2-X segregation pattern. Based on the configuration of Holiday junction resulted from loxP recombination, G2-X segregation should be predominant pattern. However, further recombination events between chromatids will increase the probability of G2-Z segregation. Therefore, the decreased G2-X segregation pattern in the presence of multiple loxP sites could be explained by additional recombination events between loxP variants prior to chromosomal segregation [21]. With this concern in mind, we tested MADM-ML not only for the overall labeling efficiency, but also for the percentage of red and green cells among total number of labeled cells as an indication of the percentage of cells undergoing G2-X segregation. We found that, in contrary to previous findings in ES cells, there was a slightly higher G2-X segregation percentage in MADM-ML compared to MADM-SL. This finding greatly alleviated our concerns of increased non-desirable G2-Z segregation due the presence of to multiple loxP sites in MADM-ML. A possible reason for the different results obtained in our study and the ES cell study [21] could be that ES and somatic cells have intrinsically biased chromosome segregation patterns [22]. 

 Using the original MADM-SL system, we attempted to model medulloblastoma with Math1-Cre. However, the tumor model had variable latency and incomplete penetrance, making it impractical for studies such as drug efficacy tests due to diminished statistical power with substantial animal-to-animal variations. Here, we showed that MADM-ML could be used in combination with Math1-Cre to produce fully penetrant medulloblastoma in a consistent timeframe, thereby making this model highly suitable for both basic research on tumor progression and pre-clinical evaluation of drug candidates. In addition to cancer modeling, MADM-ML also opens the door to widespread applications to many other research areas. For example, MADM-ML could be used to study a relatively small population of cells, such as a particular subgroup of interneurons that originates from specific precursors [23,24], to analyze how lineage relationship and genetic factors determine the proper wiring of these neurons. Furthermore, a few labs have started testing the utility of MADM-ML with CreER lines to analyze biological questions with both temporal and spatial resolution [7], which has been very difficult with MADM-SL due to extremely low recombination efficiency.

 In addition to increased recombination efficiency, MADM can still be optimized further in other aspects. Since genes amenable for MADM-mediated knockout are limited to those located between the MADM cassette and telomere on the same chromosome (Figure 1) [1], the broad application of MADM requires the engineering of all mouse chromosomes to harbor the MADM cassettes. Initial efforts have distilled important basic principles of targeted insertion of MADM cassettes into new chromosomes [7,9], which allowed the scientific community to create a number of MADM-bearing chromosomes as a collective effort. In addition to broadening the gene target range of MADM, another important modification is to use MADM not only to knockout but also to overexpress candidate genes. This has been achieved by replacing the tdTomato coding sequence with tetracycline trans-activator tTA (termed MADM-Tet) that allow the overexpression of TetO-driven transgene in MADM-labeled cells [9]. This modification enables one to use MADM to perform gain-of-function in addition to loss-of-function studies, and expands the applicability of MADM to studying genetic interactions *in vivo*.

 Recent efforts in genome sequencing and human genetics have identified a number of mutations as candidate contributing factors to a variety of human diseases [25,26]. To facilitate the understanding of the functions of these genes, the Knock Out Mouse Project (KOMP) and the European Conditional Mouse Mutagenesis Program (EUCOMM) provide invaluable mutant mouse strains for gross phenotypic analysis [27,28]. To further dissect gene function at cellular level *in vivo*, we envision that in the future, each of the mutant alleles generated by KOMP could be analyzed using the MADM system. With increased chromosomal coverage, optimization of recombination efficiency and the capacity for both gene knockout and overexpression, MADM should have ever-increasing impact on many fields of biology.

## Materials and Methods

### Animal use and care

All mice were housed and used in strict compliance with the approved protocol 11-09 by the Institutional Animal Care And Use Committee at the University of Oregon. The multiple-lox MADM11 mice will be available from The Jackson Laboratory:  GT11ML (JAX stock number 022976) and TG11ML (JAX stock number 022977).  The Jackson Laboratory also has Math1-Cre transgenic (JAX stock number 011104), Ptc-null (JAX stock number 003081), p53-null (JAX stock number 002101) and both single-lox GT11 and TG11 (JAX stock number 013749 and 013751).

### Cloning and generating MADM-ML mice

We had two important technical considerations in designing the MADM-ML targeting cassette from the MADM-SL cassette. First, there is a risk of illegitimate recombination between WT loxP and lox variants. Although Lee and Saito (1998) showed that such events rarely occur, an illegitimate recombination would create a new loxP variant that can’t recombine with any lox sites. Considering the risk, we decided to flank the added lox variant sites with FRT so we could remove them with Flippase transgene if that would become a problem. FRT sites can’t be recognized by Cre so won’t interfere with our experiments. The final configuration of multi-lox string in the MADM-ML system is FRT-lox5171-lox2272-FRT-loxP. Secondly it was difficult to synthesize two long oligonucleotide primers that encompass FRT and lox variants. Therefore, we designed two pairs of shorter primers that can be annealed and ligated together into the original MADM-SL cassette cut with Bgl II to make the MADM-ML cassette. All these primers are phosphorylated at the 5’ end for the ligation reaction to occur. The sequences of first pair of primers are GATCTCCGAAGTTCCTATTCTCTAGAAAGTATAGGAACTTCATAACTTCGTATA and

ATGTGTACTATACGAAGTTATGAAGTTCCTATACTTTCTAGAGAATAGGAACTTCGGA 
The sequences of the second pair of primers are
GTACACATTATACGAAGTTATATAACTTCGTATAGGATACTTTATACGAAGTTATG
and
GATCCATAACTTCGTATAAAGTATCCTATACGAAGTTATATAACTTCGTATA

The resulting sequence is (Bgl2 site, **FRT**, lox5171, *lox2272*, **FRT**, loxP) AGATCTCC**GAAGTTCCTATTCTCTAGAAAGTATAGGAACTTC**

ATAACTTCGTATAGTACACATTATACGAAGTTATATAACTTCGTATAGGATACTTTATACGAAGTTATGGATCTCC

**GAAGTTCCTATTCTCTAGAAAGTATAGGAACTTC**GGATCTATAACTTCGTATAGCATACATTATACGAAGTTAT

Just for comparison, the equivalent site in the original MADM-SL is (Bgl2 site, **FRT**, loxP)

#### **AGATCTCCGAAGTTCCTATTCTCTAGAAAGTATAGGAACTTCGGATCTATAACTTCGTATAGCATACATTATACGAAGTTAT**

### Mouse breeding schemes

For P5 analysis, we evaluated three genotypes of MADM mice: single loxP (SL/SL), multiple loxP (ML/ML), and mixed (ML/SL). For the SL/SL group, we generated TG11/GT11; Math1-Cre mice from the crosses of TG11/TG11; Math1-Cre/Math1-Cre with GT11/GT11 mice. For the ML/ML group, we generated TG11ML/GT11ML; Math1-Cre mice from the crosses of TG11ML/TG11ML with GT11ML/GT11ML; Math1-Cre/Math1-Cre mice. For the ML/SL group, we generated TG11/GT11ML; Math1-Cre mice from the crosses of TG11/TG11; Math1-Cre/Math1-Cre with GT11ML/GT11ML mice. P14 analysis was performed on for the SL and ML groups.

 For analysis of latency and penetrance of medulloblastoma model, we used mice derived from the cross of TG11,*p53KO*/TG11; Math1-Cre/Math1-Cre with GT11/GT11; Math1-Cre/Math1-Cre; *Ptc-KO*/WT for the MADM-SL model; and TG11ML,*p53KO*/TG11ML; Math1-Cre/Math1-Cre with GT11ML/GT11ML; Math1-Cre/Math1-Cre; *Ptc-KO*/WT for the MADM-ML model. Mice used for tumor latency and penetrance analysis were sacrificed for time course sampling, because they showed signs of illness, or for other experimental reasons. Tumor size data reported in Figure 7 was gathered from database entry of multiple experiments in our lab.

 We also used a CKO model to compare with the MADM-based medulloblastoma model, with the genotype of *Ptc-KO*/WT; *p53flox*/*p53flox*; Math1-Cre; ROSA26-floxed stop-tdTomato from the cross of *Ptc-KO*/WT; *p53flox*/*p53flox*; ROSA26-floxed stop-tdTomato with *p53flox*/*p53flox*; Math1-Cre.

### Tissue collection and immunohistochemistry

P5 brains were collected and directly fixed in ice-cold 4% paraformaldehyde for 48hrs. After three 10 min washes with 1xPBS, brains were incubated in 30% Sucrose at 4°C for 24 hrs and then embedded in Tissue Tek OCT embedding medium and stored at -80°C. P14 brains were collected after perfusion with 4% paraformaldehyde, and then processed the same as P5 brains, except for 24hr post-fix. Embedded brains were cryosectioned sagittally at 30 μm thickness, sequentially collected, 5-7 slices per slide, and five slides per brain. Sectioned slices were stained with chicken anti-GFP (1:500; Aves Labs, GFP-1020) and goat anti-myc [RFP/tdTomato is myc-tagged] (1:200; Novus, NB600-338) in PBT (PBS with 0.3% Triton-100) with 5% donkey normal serum. After multiple washing with PBT, slices were stained with secondary antibodies, including donkey anti-chicken-Cy2 (1:250; Jackson Immunoresearch) and donkey anti-goat-Alexa 555 (1:500; Invitrogen).

 Brains for tumor analysis were collected and processed with the same procedure as P14 brains. In addition to the anti-GFP and anti-myc antibodies, some tumors were stained with rabbit anti-Ki67 (1:500; Vector labs, VP-K451) and mouse anti-NeuN (1:250; Millipore, MAB377). After multiple washing with PBT, slices were stained with secondary antibodies used above, plus either donkey anti-rabbit-Alexa647or donkey anti-mouse-Alexa647 (1:250; Jackson Immunoresearch) to detect Ki67 or NeuN staining, respectively.

### Imaging and cell counting

Immuno-stained sections were examined and photographed using an Olympus FV-1000 upright laser confocal microscope. Images were taken using the 20x objective, line sequential and kalman at 512x512 pixels. P5 brains were analyzed in a defined square, (10x10cm as viewed on a computer screen) between the fifth and sixth folia at the base of the sulcus, and only cells in the EGL were counted. P14 brains were analyzed in a defined square (5x5cm as viewed on a computer screen) at the base of the third folia, and only cells in the IGL were counted. To ensure systematic sampling, slices at three distinct medial to lateral planes from each brain were used for cell counting. For gross tumor imaging, half brains cut sagittally were imaged with an Olympus MVX-10 macroscope for tumor size assessment.

### Statistical Analysis

Statistical analysis was performed using MatLab software, GraphPad Prism and Microsoft Excel. Cell counts from all three slices of each brain were summed for each data point. P5 data were analyzed using one-way ANOVA, and Multiple Comparisons using the Tukey HSD test (n=4 for SL/SL, n=5 for ML/ML, and n=5 ML/SL). The average total labeled cells (sum of all 3 brain slices) counted for SL/SL=86.29, ML/ML=831.22, and ML/SL=48.94. P14 data were analyzed using an independent samples t-test with unequal variance (n=5 for SL/SL and n=7 for ML/ML groups). The average total labeled cells counted for SL/SL=317.6 and ML/ML=880.62.

## Supporting Information

Figure S1 **Potential uses for MADM.** (A) Illustration of the application of MADM for studying cell-autonomous gene functions in cellular morphogenesis. Illustrated is a hypothetical example of how MADM could reveal the subcellular details of dendritic morphology to demonstrate the role of a given gene in dendritic branch formation. (B) Illustration of how MADM can be used to trace lineages and study the fate of both green and red cell lineages. Hypothetically, in a tissue consisting of two cell types of unknown lineage relationship, MADM can determine if the two cell types are generated directly from a multipotent stem cell or indirectly from unipotent intermediate progenitor cells. If the pattern shown were observed, one would conclude that the latter is true.
(TIF)

Figure S2 **Math1-Cre labels a small fraction of GNPs with MADM.** At P5, Math1-Cre labels almost all GNPs with a floxed-stop tdTomato Cre reporter (middle panel, in comparison to DAPI staining in the left panel). However, the labeling of MADM with Math1-Cre is very sparse (right panel). Scale bar: 20um.
(TIF)
