# Supplementary figures and images for: MADM-ML, a Mouse Genetic Mosaic System with Increased Clonal Efficiency
Source: PLoS One. 2013 Oct 15;8(10):e77672. doi: 10.1371/journal.pone.0077672 (PMC3797059; doi:10.1371/journal.pone.0077672)

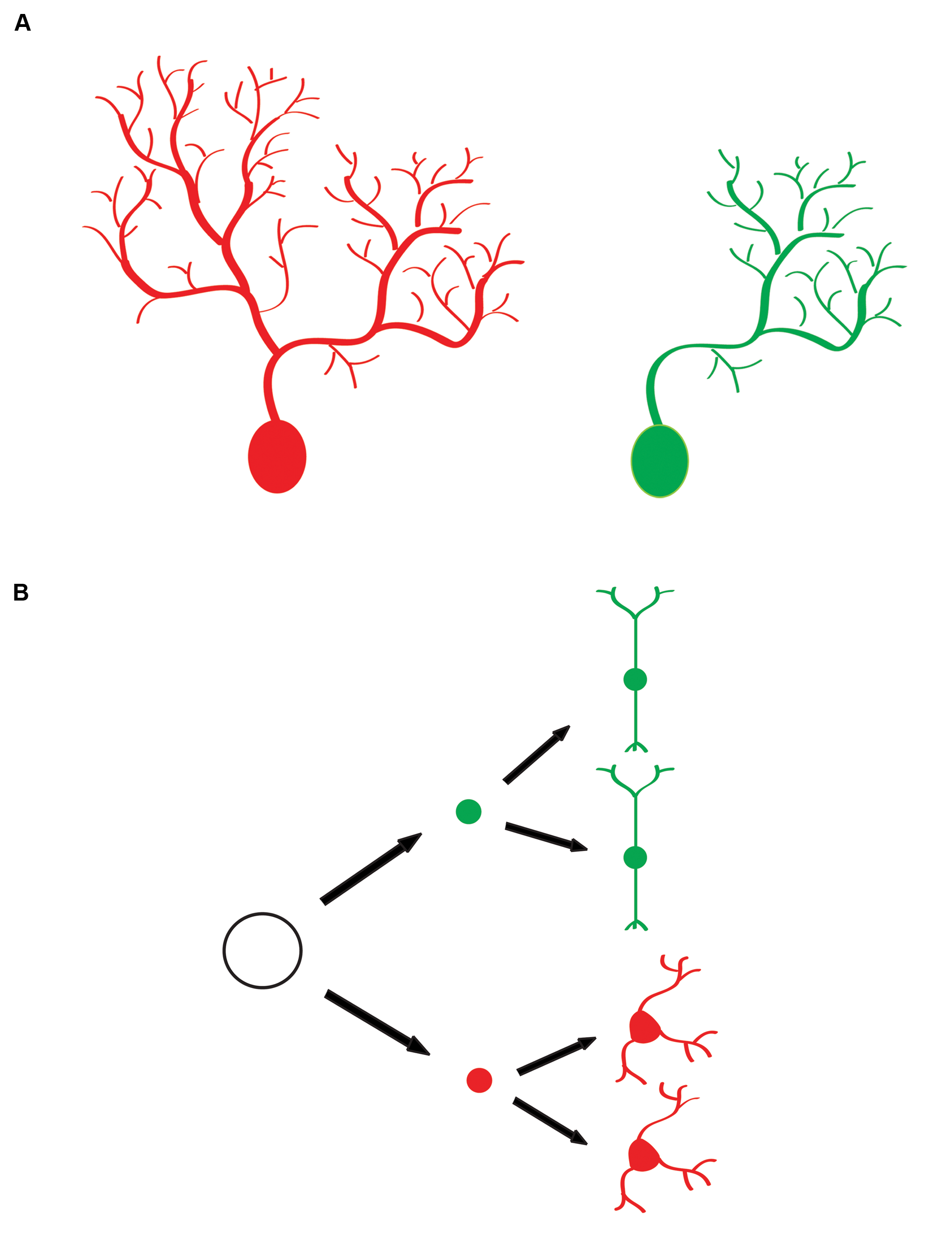

Supplement: Figure S1 — Potential uses for MADM. (A) Illustration of the application of MADM for studying cell-autonomous gene functions in cellular morphogenesis. Illustrated is a hypothetical example of how MADM could reveal the subcellular details of dendritic morphology to demonstrate the role of a given gene in dendritic branch formation. (B) Illustration of how MADM can be used to trace lineages and study the fate of both green and red cell lineages. Hypothetically, in a tissue consisting of two cell types of unknown lineage relationship, MADM can determine if the two cell types are generated directly from a multipotent stem cell or indirectly from unipotent intermediate progenitor cells. If the pattern shown were observed, one would conclude that the latter is true. (TIF) [file pone.0077672.s001.tif]

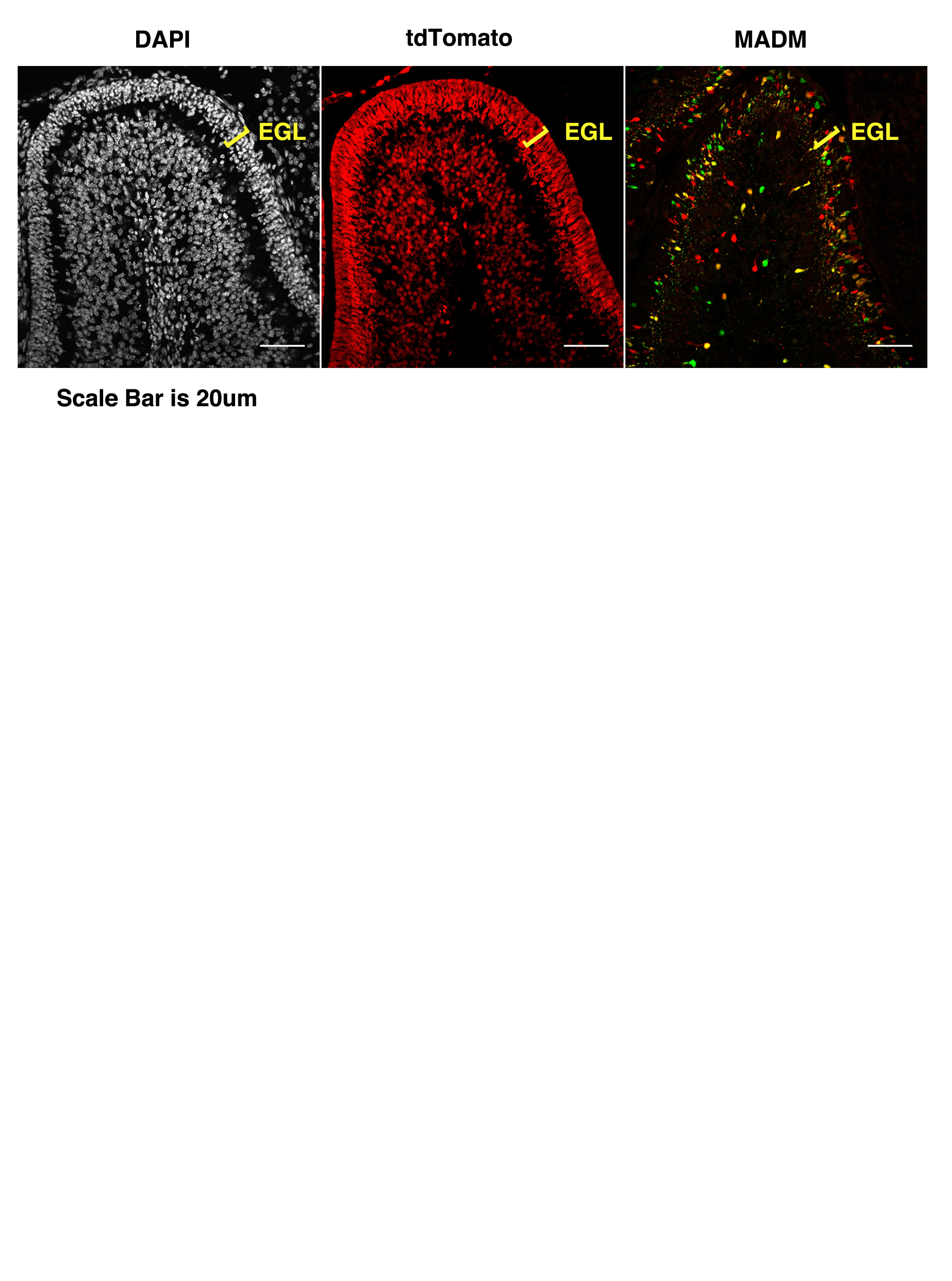

Supplement: Figure S2 — Math1-Cre labels a small fraction of GNPs with MADM. At P5, Math1-Cre labels almost all GNPs with a floxed-stop tdTomato Cre reporter (middle panel, in comparison to DAPI staining in the left panel). However, the labeling of MADM with Math1-Cre is very sparse (right panel). Scale bar: 20um. (TIF) [file pone.0077672.s002.tif]
